# In-plane gate graphene transistor with epitaxially grown molybdenum disulfide passivation layers

**DOI:** 10.1038/s41598-023-36405-9

**Published:** 2023-06-06

**Authors:** Po-Cheng Tsai, Chun-Wei Huang, Shoou-Jinn Chang, Shu-Wei Chang, Shih-Yen Lin

**Affiliations:** 1 Research Center for Applied Sciences, Academia Sinica, 128 Academia Road, Section 2, Nankang, Taipei, 11529 Taiwan; 2 Institute of Microelectronics, National Cheng Kung University, No.1, University Road, Tainan City, 701 Taiwan; 3 Department of Electrical Engineering, National Cheng Kung University, No.1, University Road, Tainan City, 701 Taiwan; 4 Department of Photonics, National Yang Ming Chiao Tung University, 1001 University Road, Hsin-chu, 30010 Taiwan

**Keywords:** Electronic devices, Electronic devices

## Abstract

We demonstrate in-plane gate transistors based on the molybdenum disulfide (MoS_2_)/graphene hetero-structure. The graphene works as channels while MoS_2_ functions as passivation layers. The weak hysteresis of the device suggests that the MoS_2_ layer can effectively passivate the graphene channel. The characteristics of devices with and without removal of MoS_2_ between electrodes and graphene are also compared. The device with direct electrode/graphene contact shows a reduced contact resistance, increased drain current, and enhanced field-effect mobility. The higher field-effect mobility than that obtained through Hall measurement indicates that more carriers are present in the channel, rendering it more conductive.

## Introduction

The high mobility and bright luminescence of two-dimensional (2D) materials are their major advantages in device applications^1–6^. Despite its zero-bandgap nature, the high mobility of the first discovered 2D material, graphene, have demonstrated its potential in the application of radio-frequency devices^7–11^. On the hand, since the unique material characteristics of 2D materials can be observed in a few atomic layers, devices with ultra-thin bodies can be fabricated on these materials, making them a promising candidate for advanced electronics with reduced linewidth^12–15^. However, the characteristics of thin body also hinder the practical usage of 2D materials. With an active region down to nanoscale, little room is left for 2D channels vertically. Since the top gate scheme is the most common device architecture adopted for field-effect transistors (FETs) in industry, a poor dielectric/2D-material interface can easily degrade the performance of thin 2D transistors^16–18^. One possible approach to solve this problem is to use dielectric 2D materials such as hexagonal boron nitride (h-BN) as the interface layers to the SiO_2_ dielectrics. Due to the atomically flat surface and wide bandgap value of h-BN, superior device performances are obtained for such bottom-gate graphene transistors^19,20^. The other possible solution may be the adoption of different device architecture without dielectric/2D-material interfaces. In previous works, it has been shown that transistors with in-plane gates can be realized on compound semiconductors^21,22^. This device architecture may lower the influence of dielectric/2D-material interfaces on 2D channels.

However, without top covering, the channel is exposed to the atmosphere. Water or oxygen molecules in air may attach to the 2D channel and degrade the device performance. A passivation layer is still necessary to protect the channel from environments. Without extra chemical bonding, passivation based on another 2D material may bring the least impact to the 2D channel. Similar to being interfacial layers on the SiO_2_, h-BN is also adopted as the passivation layers to 2D material devices^23,24^. However, due to the high growth temperatures of h-BN, sequential mechanical exfoliations of different 2D materials instead of epitaxial growth are usually adopted for such hetero-structures, which may introduce additional contaminations to the 2D material interfaces. Nevertheless, the results still demonstrated that a less conductive 2D material may act as the passivation layer for the 2D channel. Together with in-plane gates, the 2D channel passivated by epitaxially grown 2D materials may exhibit optimized performances. In this work, we fabricated and characterized in-plane gate transistors (IPGTs) based on molybdenum disulfide (MoS_2_)/graphene hetero-structure^25–27^. IPGTs with a 300 nm gate-channel separation were fabricated on the MoS_2_/graphene film using the e-beam lithography. To further improve the device performances, we utilized atomic layer etching (ALE) technique to remove the MoS_2_ beneath the source/drain electrodes. In this way, the electrode metal would contact the graphene channel directly. The MoS_2_ passivation can effectively isolate the graphene channel from the environment. A field-effect mobility of the device similar to that from Hall measurements indicates that the device performance is well maintained using in-plane gates.

## Results and discussions

### In-plane gate transistors with standalone graphene

**Figure 1:**
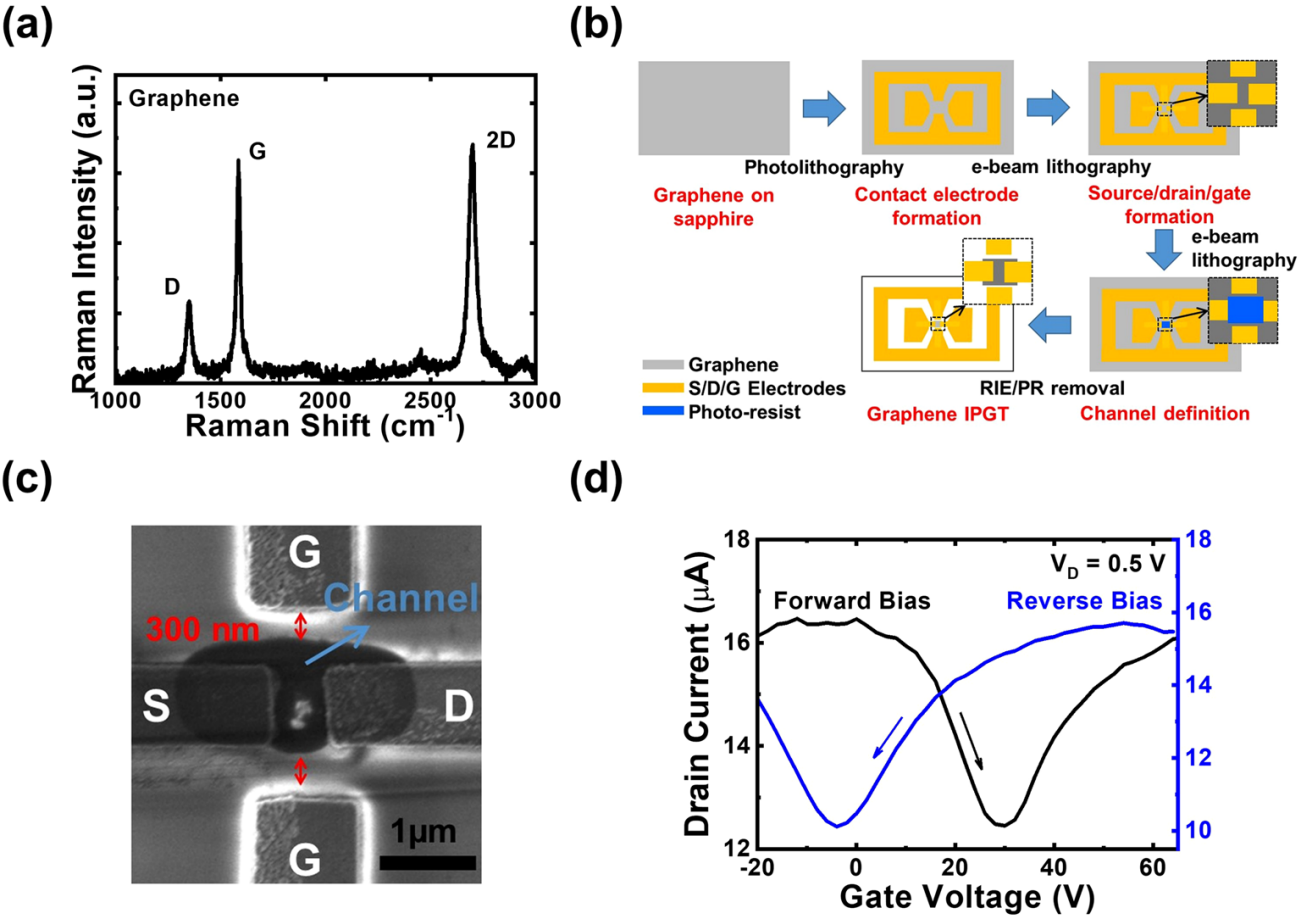
(**a**) The Raman spectra of the graphene film grown directly on sapphire substrates. (**b**) The fabrication steps of the graphene IPGTs. (**c**) The SEM image of the device. (**d**) The transfer curve of the in-plane gate transistors measured under forward and reverse gate biases at ${V}_{\mathrm{DS}}$ = 0.5 V.

For demonstrating the feasibility of 2D materials on the architecture of in-plane gate transistors (IPGTs), a graphene film grown directly on sapphire substrates is prepared. The Raman spectrum of the sample is shown in Fig. 1a. The ratio of D/G peak is around 0.4, indicating that while a continuous graphene film could be grown directly on sapphire substrates using CVD, a limited graphene grain size and non-negligible defect density featured by the D peak were still present in the graphene film. The detailed discussions on the growth and formation mechanisms of the graphene films grown directly on sapphire substrates are demonstrated elsewhere^25^. Nevertheless, compared with the counterparts grown on copper foils, the graphene films grown on sapphire substrates require no transferring processes and can further simplify the device fabrication. Therefore, this growth scheme is suitable for the architecture of IPGTs. The fabrication steps of the in-plane gate graphene transistor are illustrated in Fig. 1b. The large-area electrodes were fabricated using the standard photolithography. After that, the e-beam lithography was utilized to fabricate the IPGT. The scanning electron microscope (SEM) image of the device near the channel is shown in Fig. 1c. After the device fabrication procedure, an in-plane gate graphene transistor with channel width/length 500/500 nm and the gate-channel separation 300 nm can be fabricated. The voltage-current transfer curves of the device under forward and reverse biases at ${V}_{\mathrm{DS}}=$ 0.5 V are shown in Fig. 1c. Standard characteristics of graphene transistor with the Dirac point located at about 30 V gate biases are observed for the device under forward bias condition. Since we could not observe current modulation with gate biases for the device with larger gate separation 1000 nm, the transistor performances of the device should be attributed to the smaller gate separation 300 nm and therefore, higher electric fields between gate and source electrodes. Further investigation is still required in the future to clarify the operation mechanisms of the device. We use the following formula to estimate the field-effect mobility ${\mu }_{\mathrm{FET}}$ at ${V}_{\mathrm{DS}}=$ 0.5 V (linear regime):

1 $$ \mu_{{{\text{FET}}}} = \frac{1}{g}\frac{1}{{V_{{{\text{DS}}}} }}\frac{d}{\varepsilon }\frac{L}{W}\frac{{{\text{d}}I_{{{\text{DS}}}} }}{{{\text{d}}V_{{{\text{GS}}}} }}, $$

where $d$ is the gate-to-channel separation; $\varepsilon$ is the dielectric constant of air; $L$ and $W$ are the channel length and width, respectively; and the factor $g=2$ accounts for the number of in-plane gates at two sides of the channel. The derived hole and electron mobilities of the device are 90.0 and 77.0 cm^2^ V^−1^ s^−1^, respectively. The field-effect hole mobility is slightly lower than the value commonly observed for the directly grown graphene films via the Hall measurement (p-type, 100–200 cm^2^ V^−1^ s^−1^), which may be attributed to the water or oxygen attachment from the environment. The results suggest that although the in-plane device architecture may avoid the influence of dielectric layers on the graphene channel, the influence from the atmospheric condition is un-avoided with the graphene channel exposed to air. On the other hand, when the device is operated under reverse bias condition, significant Dirac point shift from 30 to − 4.0 V is also observed in Fig. 1c. It has been demonstrated in previous publications that the Dirac point of bottom-gate graphene transistor from positive to close to zero gate voltage with increasing measurement temperatures up to 100 ^o^C^28^. The results demonstrated that the water molecules in the atmospheric condition attach to the graphene surface and influence the device performances. In this case, when the graphene transistor is under operation, the dipoles of absorbed water molecules are oriented along the direction of applied gate bias, which increases the local electric field near dipoles and therefore, increase the carrier density in the graphene channel^29^. In this case, a clear hysteresis loop is observed for the in-plane gate graphene transistor without a passivation layer.

### In-plane gate graphene transistors with MoS_2_ passivation layers

**Figure 2:**
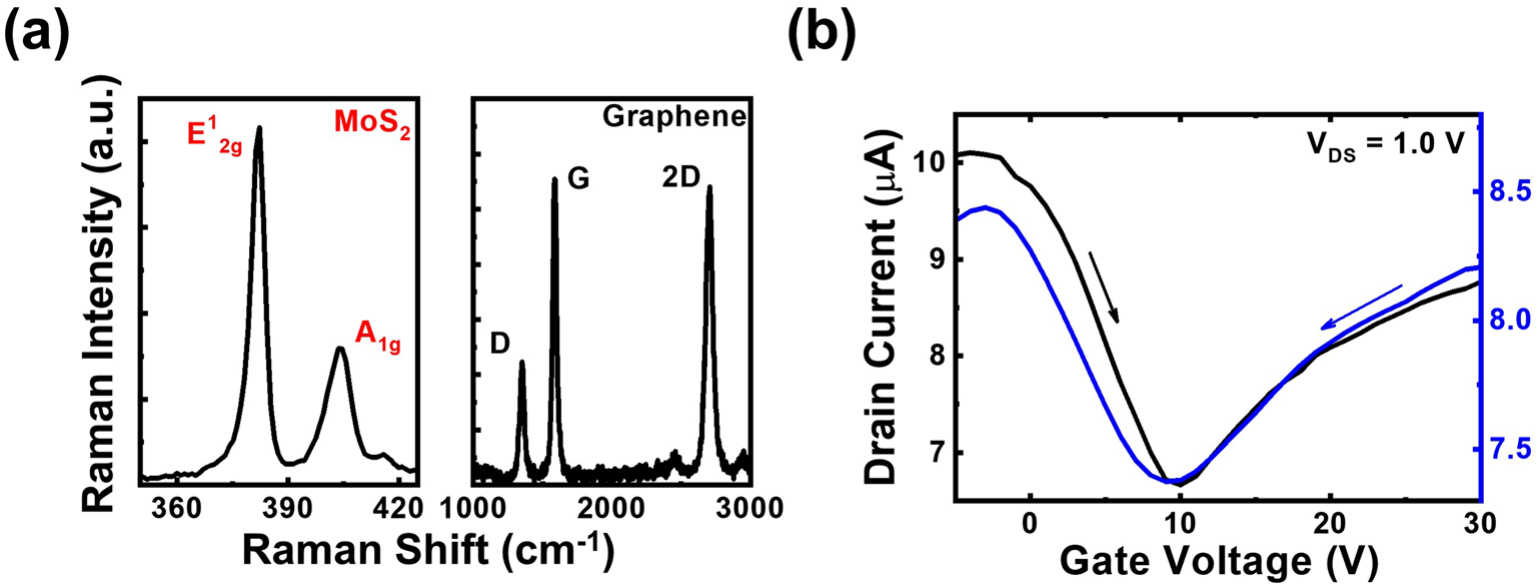
(**a**) The Raman spectra of MoS_2_/graphene hetero-structure and (**b**) the transfer curve of the MoS_2_/graphene IPGT at ${V}_{\mathrm{DS}}$= 1.0 V.

The passivation layer is a possible solution to prevent the attachment of water or gas molecules to the graphene channel^28^. Since dielectric materials such as Al_2_O_3_ may bring additional influence to the graphene channel and do not conform to the concept behind IPGTs, the most promising candidate for passivation layers should be other less conductive 2D material layers. In previous publication, we have demonstrated that by sulfurizing pre-deposited Mo films, MoS_2_ layers could be grown on graphene surfaces^30^. However, since the Ar plasma in the radio-frequency sputtering system may bring additional damage to graphene films, we formed the MoS_2_/graphene hetero-structure by sulfurizing the MoO_3_ film deposited with the thermal evaporator. The preparation procedure of the MoS_2_/graphene hetero-structure is disclosed in the “Methods” section. The Raman spectra of the MoS_2_/graphene hetero-structure with different measurement ranges corresponding to the characteristic Raman peaks of graphene and MoS_2_, respectively, are shown in Fig. 2a. The observation of both the graphene and MoS_2_ Raman characteristic peaks indicates that the MoS_2_/graphene hetero-structure is formed after the sequential growth of graphene and MoS_2_. The difference ∆*k* between the two Raman peaks of MoS_2_ is 21.3 cm^−1^, indicating the presence of bi-layer MoS_2_ after the sulfurization procedure^31^. On the other hand, the similar D/G peak ratios before and after the sulfurization procedure of the deposited MoO_3_ film (around 0.4) suggest that the possible damages introduced during either the MoO_3_ deposition or the sulfurization procedures are almost negligible. Although the electrical properties of MoS_2_ will be degraded in air^32^ in the IPGT architecture, it is not a concern here since the MoS_2_ layers are the passivation layers instead of channels. Therefore, the degradation of the MoS_2_ layers in the ambient condition may not influence the performance of the device. Following the similar device fabrication of standalone graphene IPGTs (Fig. 1b), MoS_2_/graphene IPGTs with gate separations of 300 nm were fabricated. The transfer curves of the device under forward and reverse biases at ${V}_{\mathrm{DS}}=$ 1.0 V are shown in Fig. 2b. Similar with the graphene IPGT, the device behaves as a typical graphene transistor. The Dirac point at a positive ${V}_{\mathrm{GS}}=$ 10.0 V indicates a p-type graphene channel under forward biases. The weak hysteresis on the transfer curves suggests that the MoS_2_ layer can effectively protect the channel from contaminants in environments. However, compared to IPGT with un-passivated graphene, much lower drain currents are observed for the MoS_2_-passivated device. Therefore, a higher drain voltage 1.0 V is adopted for the measurement of the MoS_2_-passivated device. By using Eq. (1), the derived hole and electron mobilities of the device are 61.0 and 31.0 cm^2^ V^−1^ s^−1^, respectively. Compared with the hole mobility 100–200 cm^2^ V^−1^ s^−1^ of the graphene film on sapphire from Hall measurements, the field-effect mobility is lower. The derived field-effect mobility values are also lower than the standalone graphene IPGTs discussed in the last section. The results may be attributed to the high contact resistance at the electrode/MoS_2_ interfaces which hinder the supply of carriers as the gate voltage is changed^33^.

### Direct electrode contact with the graphene channel through ALE of MoS_2_

**Figure 3:**
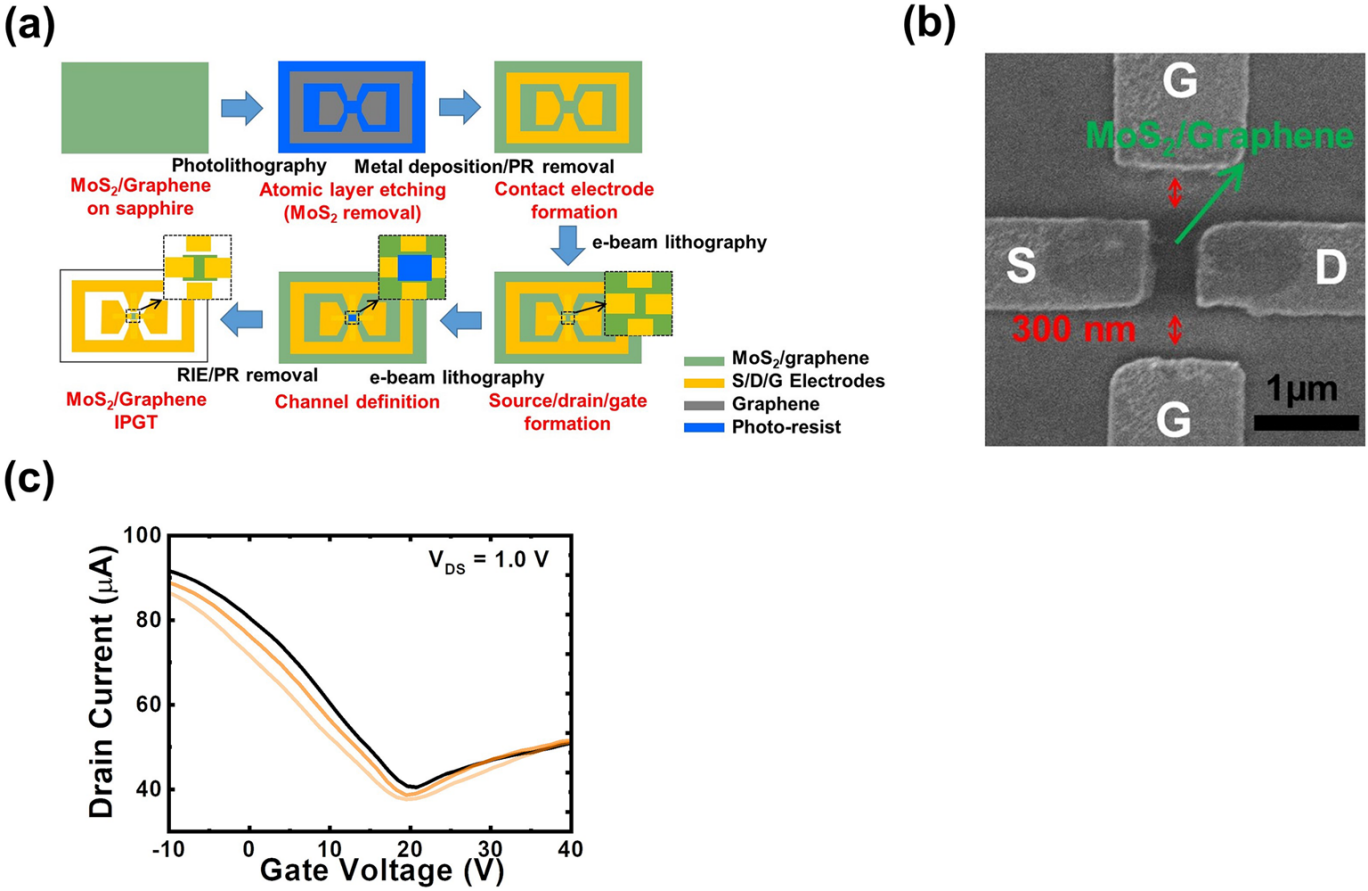
(**a**) The fabrication steps, (**b**) the SEM image and (**c**) transfer curve at *V*_DS_ = 1.0 V of the IPGT with MoS_2_ beneath electrodes removed. The transfer curves of the other two MoS_2_/graphene IPGTs are also shown as colored lines in (**c**).

In one previous publication, we have demonstrated that the atomic layer etching (ALE) of multi-layer MoS_2_ can be achieved by using low-power oxygen plasma treatment and following water dipping^34^. To examine the effect of contact resistance at the electrode/MoS_2_ interfaces, we additionally removed the MoS_2_ beneath electrodes by using the ALE technique before the electrode deposition. Figure 3a shows the corresponding fabrication steps. Since there are two layers of MoS_2_ on graphene, two ALEs were performed to remove the MoS_2_ below the electrode. The SEM image of the device is shown in Fig. 3b. To demonstrate the precise atomic etching procedure, the other sample with the same bi-layer MoS_2_/graphene hetero-structure is prepared. The Raman spectra of the sample showing the evolution of MoS_2_ characteristic Raman peaks with ALE times are shown in Fig. S1 of the supplementary material. The characteristic Raman peaks of graphene after two-time ALEs are also shown in Fig. S2. After that, the device fabrication followed that without the removal of MoS_2_. The transfer curve of the new device at ${V}_{\mathrm{DS}}=$ 1.0 V is shown in Fig. 3c. The Dirac point of this device is around ${V}_{\mathrm{GS}}=$ 20 V. Also shown in the figure is a significant current enhancement as compared to that in Fig. 2b in the same range of gate voltage. This indicates that with the removal of MoS_2_ beneath electrodes, the contact resistance becomes significantly lower. Using Eq. (1), we estimate the hole and electron mobilities of the device as 328.0 and 187.0 cm^2^ V^−1^ s^−1^, respectively. To demonstrate the reproducibility of the device performances, two more MoS_2_/graphene IPGTs are fabricated following the same procedure shown in Fig. 3a. The transfer curves of the two devices are also shown as colored lines in Fig. 3c. The similar device performances have demonstrated the reproducibility of the IPGT architecture.

In addition to the current enhancement due to the more significant accumulation of the carrier in the channel, the field-effect mobilities become 2–3 times higher than the Hall-effect ones from graphene grown on the sapphire. We note that the mobility extracted from the Hall measurement reflects the transport properties without significantly altering the intrinsic carrier density of graphene on sapphire. As a result, without being screened at low-density level, the scatterings due to lattice imperfections such as charged impurities or structural defects can all impede carrier drifting. In contrast, after the issue of contact resistance is resolved, the in-plane gates may easily attract more carriers into the graphene channel. The Coulomb effect from these additionally provided carriers can screen the interactions between lattice imperfections and drifted carriers. The scattering in the channel is screened more in the presence of the higher carrier density. Therefore, by increasing the carrier density through gating, not only the drain current but also the mobility is enhanced.

### Photo-responses of the MoS_2_/graphene IPGTs

**Figure 4:**
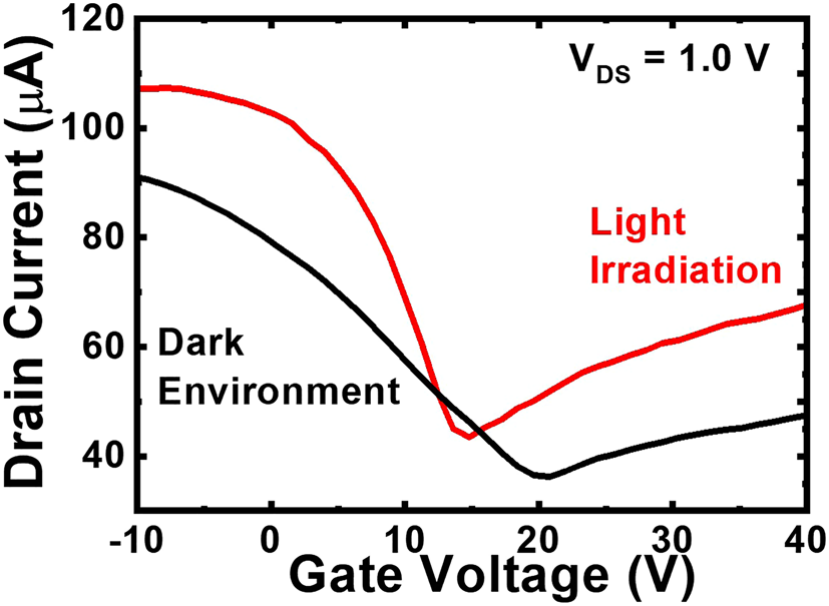
The transfer curves of three devices without MoS_2_ beneath electrodes at *V*_DS_ = 1.0 V under the dark and light-irradiation conditions.

The transfer curves of the device under dark and light-irradiation conditions are shown in Fig. 4. The light source is a white light source equipped with the microscope of the probe station. The Dirac point shift from 20.0 to 15.0 V suggests that photo-excited electrons in the MoS_2_ layer are attracted by the positive drain voltage to the graphene channel and change the Fermi level^35^. The results revealed that with more complicated 2D material structures such as MoS_2_/graphene hetero-structures, different device applications such as photo-transistors can be demonstrated. With Eq. (1), the derived hole and electron mobilities of the device reach 1210.0 and 205.0 cm^2^ V^−1^ s^−1^, respectively, under light illumination. The increment of field-effect mobility may be also attributed to the enhanced screening effect mentioned earlier in the presence of photo-excited carriers. On the other hand, the potential of scalability and reproducibility of the device is important for practical applications. The transfer curves of two more devices with the same device architecture under dark and light-irradiation conditions are shown in Fig. S2 of the supplementary material. The similar device performances have demonstrated that the scalability and reproducibility of the in-plane gate graphene transistors can be achieved through the fabrication procedure shown in Fig. 3a.

## Conclusion

With the graphene on sapphire as the new substrate, we grew MoS_2_/graphene hetero-structures by sulfurizing the MoO_3_ film deposited on graphene. IPGTs were fabricated on the MoS_2_/graphene hetero-structure. By using MoS_2_ as the passivation layer, a weak hysteresis on the transfer curves is observed for the IPGT, which suggests that the MoS_2_ layer can effectively protect the graphene channel from contaminants in environments. After the removal of MoS_2_ beneath electrodes, the direct contact between electrodes and graphene shows reduced contact resistance. This increases the field-effect mobility and drain current of the device. The architecture of IPGT provides an alternate approach for the fabrication of 2D transistors with the less non-ideal interface effect.

## Methods

The graphene films were grown by using the chemical vapor deposition (CVD) in a hot furnace at 1100 °C with the ethane precursor and Ar/H_2_ carrier gas directly on sapphire substrates^25^. With the graphene/sapphire sample as the new substrate, 1.0 nm thick molybdenum trioxide (MoO_3_) was deposited on the graphene/sapphire substrate using the thermal evaporation. The sample was then sulfurized in a furnace at 850 °C to convert MoO_3_ into MoS_2_^30^. After the definition of large-area contact electrodes through typical photolithography, IPGTs were fabricated with the aid of e-beam lithography. The electrodes composed of 50 nm Au/10 nm Ti were deposited with the e-beam evaporator. For the removal of MoS_2_ beneath the electrodes, we used a customer-designed low-pressure RF oxygen plasma system to perform ALE. The plasma power was kept at 20 W, and the background pressure was maintained at 0.4 Torr with a 30 sccm oxygen gas flow during the removal process. The etching time was 10 s. After this, the sample was dipped into deionized water for 10 s to detach the topmost oxidized MoS_2_ layer^34^. The current–voltage curves were taken with probes equipped with a Keithley 2636B system. Raman spectra were collected using a HORIBA Jobin Yvon HR800UV Raman spectroscopy system equipped with a 488 nm laser. The room-temperature Hall mobility was obtained with the Ecopia HMS-5000 Hall effect measurement system.

## Supplementary Information

Supplementary Information.

## Data Availability

All data generated or analysed during this study are included in this published article (and its Supplementary Information files).
