# Supplementary material for: In-plane gate graphene transistor with epitaxially grown molybdenum disulfide passivation layers
Source: Sci Rep. 2023 Jun 6;13:9197. doi: 10.1038/s41598-023-36405-9 (PMC10244327; doi:10.1038/s41598-023-36405-9)
Supplement: Supplementary file 1 — Supplementary Information. [file 41598_2023_36405_MOESM1_ESM.pdf]

In-plane Gate Graphene Transistor with Epitaxially Grown Molybdenum Disulfide Passivation Layers

Po-Cheng Tsai[1], Chun-Wei Huang[2], Shoou-Jinn Chang[2, 3], Shu-Wei Chang[1, 4, *] and Shih-Yen Lin[1, 3, *]

[1]Research Center for Applied Sciences, Academia Sinica, 128 Academia Road, Section 2, Nankang, Taipei, 11529, Taiwan

[2]Institute of Microelectronics, National Cheng Kung University, No.1, University Road, Tainan City 701, Taiwan

[3]Department of Electrical Engineering, National Cheng Kung University, No.1, University Road, Tainan City 701, Taiwan

[4]Department of Photonics, National Yang Ming Chiao Tung University, 1001 University Road, Hsinchu 30010, Taiwan

*Corresponding author, electronic mail: swchang@sinica.edu.tw, shihyen@gate.sinica.edu.tw

We have prepared another sample with bi-layer MoS_2 grown on the graphene surface following the same growth procedure discussed in the current manuscript. The Raman spectra of the as-grown sample and the sample after one- and two- times of ALE procedures discussed in the current manuscript are shown in Fig. S1. As shown in the figure, the Δk value will decrease from 22.0 to 21.0 cm^{-1} after a one-time ALE procedure. After two times of ALE procedures, the characteristic Raman peaks of MoS_2 will disappear. On the other hand, we did not observe a significant increase in the D/G peak ratio of the graphene Raman signals after two times of ALE procedures. The results exhibit that the low-power oxygen plasma did not introduce a severe damage to the graphene film for direct metal contact. Therefore, we believe that the main mechanism responsible for the device performance enhancement of the MoS_2-passivated graphene in-plane gate transistor with additional ALE procedures before the metal deposition is still the removal of the MoS_2 layers such that a reduced contact resistance is obtained with electrodes contacting directly to the graphene layer. However, it is possible that the minor oxygen plasma treatment to the graphene layer underneath the electrodes may also help to reduce the contact resistance at the metal/graphene interface. Further investigation is still required to clarify this point.

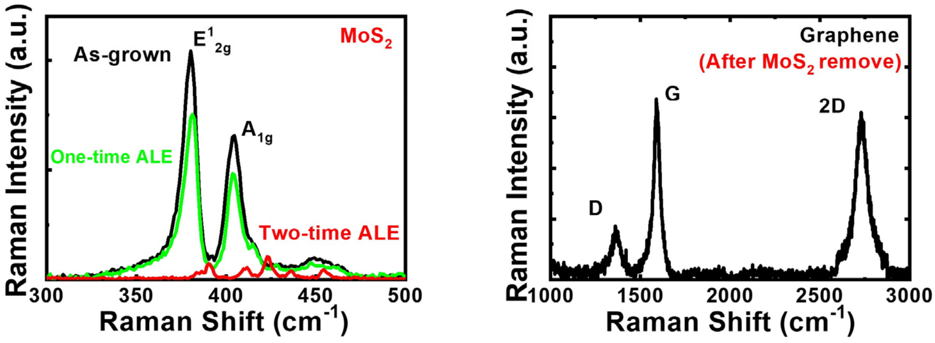

Fig. S1. The Raman spectra of the as-grown sample and the sample after one- and two-times of ALE procedures showing the MoS_2 characteristic Raman peaks (left figure). The Raman spectrum of the sample after two-time ALE procedures showing the graphene characteristic Raman peaks.

To demonstrate the reproducibility of the in-plane gate graphene transistors, the transfer curves of two more devices with the same device architecture as discussed in the current manuscript (with the MoS_2 passivation layer and two ALE procedures before the S/D metal deposition) under dark and light-irradiation conditions are shown in Figure S2. The similar device performances have demonstrated that the scalability and reproducibility of the in-plane gate graphene transistors can be achieved through the fabrication procedure shown in Fig. 3 (a).

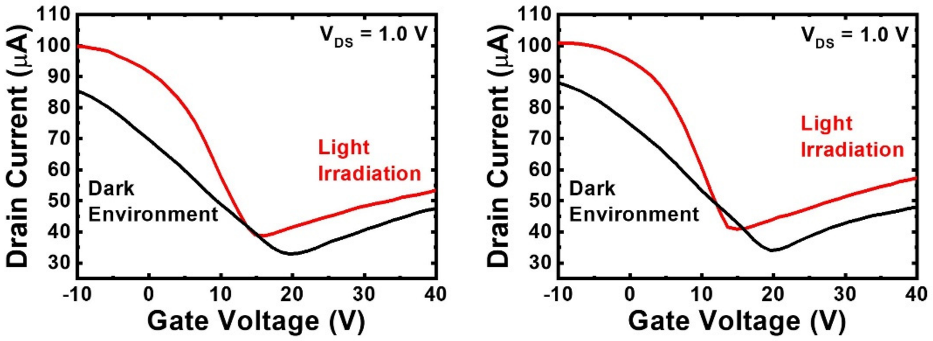

Fig. S2. The transfer curves of two in-plane gate graphene transistors measured under dark and light- irradiation conditions at V_{DS} = 1.0 V.
